# Complete Genome Sequence of Influenza Virus H9N2 Associated with a Fatal Outbreak among Chickens in Dubai

**DOI:** 10.1128/genomeA.00752-16

**Published:** 2016-08-18

**Authors:** Siu-Ying Lau, Sunitha Joseph, Kwok-Hung Chan, Honglin Chen, Nissy Annie Gerogy Patteril, Shyna K. Elizabeth, Rubeena Muhammed, Vijay Baskar, Susanna K. P. Lau, Joerg Kinne, Ulrich Wernery, Patrick C. Y. Woo

**Affiliations:** a Department of Microbiology, The University of Hong Kong, Hong Kong, China; b Central Veterinary Research Laboratory, Dubai, United Arab Emirates; c State Key Laboratory of Emerging Infectious Diseases, The University of Hong Kong, Hong Kong, China; d Research Centre of Infection and Immunology, The University of Hong Kong, Hong Kong, China; e Carol Yu Centre for Infection, The University of Hong Kong, Hong Kong, China; f Collaborative Innovation Center for Diagnosis and Treatment of Infectious Diseases, The University of Hong Kong, Hong Kong, China

## Abstract

We report the complete genome sequence of influenza virus H9N2 associated with a fatal outbreak among chickens in Dubai. All segments are clustered with avian H9N2 viruses circulating in the Middle East but distinct from those in southeast Asia. It is not a reassortant virus or transmitted from other regions.

## GENOME ANNOUNCEMENT

Influenza virus outbreaks due to H5N1, H5N6, H5N2, and H7 subtypes are common in poultry and have led to huge economic loss worldwide and are occasionally associated with human infections (1–3). However, outbreaks associated with influenza virus H9N2 have been extremely uncommon over the two decades since this subtype adapted and has been prevalent in chicken. Chickens infected with H9N2 virus are asymptomatic and egg production may be affected. In a previous report of an influenza virus H9N2 outbreak in chickens in the Republic of Korea, comparative genome analysis showed high sequence similarity to genes of reassortant H9N2 viruses in slaughterhouses and live bird markets (4).

In November 2015, ~30 chickens died in a chicken farm in Dubai, the United Arab Emirates, and all the chickens in the farm were culled. The farm has 20,000 layers of different breeds of leghorn chickens around 60 weeks old from Saudi Arabia. The farm consisted of four blocks with four houses per block, with the farm completely fenced. Altogether there were 19 staff members with one looking after the chickens in each house. There was no contact between chickens in the farm and other chickens or birds. The chickens received the influenza virus H9N2 vaccine and 85% of the chickens were H9 antibody-positive. Influenza virus A/Chicken/Dubai/D2506.A/2015 was isolated from the lung fluid of the chickens using 9 to 11-day-old embryonated chicken eggs and complete genome sequencing showed H9N2 subtype. In this article, we report the complete genome sequence of the influenza virus H9N2 strain associated with this outbreak.

Viral RNA was extracted using a QIAamp Viral RNA minikit (QIAGEN) and cDNAs were synthesized by reverse transcription (TaKaRa). The full length viral genome was sequenced as previously described (5). Sequence fragments were assembled with Lasergene (version 6.0; DNASTAR) then aligned and residue analyzed using BioEdit version 7. Sequences of avian H9N2 virus were used for comparison. A/Chicken/Dubai/D2506.A/2015 contains no known virulent elements in the hemagglutinin, such as the avian type 627E residue observed in multiple basic motif H5 or H7 subtypes and PB2. Using sequences of other H9N2 viruses extracted from GenBank, phylogenetic trees were constructed using the neighbor-joining method with the Tamura-Nei model of nucleotide substitutions in the MEGA program (version 5.05). It is shown that all segments are clustered with avian H9N2 viruses circulating in the Middle East but distinct from H9N2 viruses in southeast Asian countries (6, 7). Based on these findings, we conclude that A/Chicken/Dubai/D2506.A/2015 is not a reassortant virus and not transmitted from other regions. While no known virulent element was identified in the viral genome from A/Chicken/Dubai/D2506.A/2015, several mutations were observed in HA, NA, and other internal genes. It is possible that there are uncharacterized virulent mutations contributing to the outbreak in chickens. Pathogenicity in poultry and other animal models may be necessary to assess the pathogenic properties of this variant. Surveillance for any potential virulence change in H9N2 virus is warranted.

### Accession number(s).

This genome has been deposited at DDBJ/EMBL/GenBank under accession no. KX351195 to KX351202.

## References

[1] Yuen KY, Chan PK, Peiris M, Tsang DN, Que TL, Shortridge KF, Cheung PT, To WK, Ho ET, Sung R, Cheng AF 1998 Clinical features and rapid viral diagnosis of human disease associated with avian influenza A H5N1 virus. Lancet 351:467–471. doi:10.1016/S0140-6736(98)01182-9. 9482437

[2] Chen Y, Liang W, Yang S, Wu N, Gao H, Sheng J, Yao H, Wo J, Fang Q, Cui D, Li Y, Yao X, Zhang Y, Wu H, Zheng S, Diao H, Xia S, Zhang Y, Chan K-H, Tsoi H-W, Teng JL-L, Song W, Wang P, Lau S-Y, Zheng M, Chan JF-W, To KK-W, Chen H, Li L, Yuen K-Y 2013 Human infections with the emerging avian influenza A H7N9 virus from wet market poultry: clinical analysis and characterisation of viral genome. Lancet 381:1916–1925. doi:10.1016/S0140-6736(13)60903-4. 23623390 PMC7134567

[3] Yang Z, Mok CKP, Peiris JSM, Zhong N 2015 Human infection with a novel avian influenza A(H5N6) virus. N Engl J Med 373:487–489. doi:10.1056/NEJMc1502983. 26222578

[4] Lee YN, Lee DH, Park JK, Lim TH, Youn HN, Yuk SS, Lee YJ, Mo IP, Sung HW, Lee JB, Park SY, Choi IS, Song CS 2011 Isolation and characterization of a novel H9N2 influenza virus in Korean native chicken farm. Avian Dis 55:724–727. doi:10.1637/9774-050911-Case.1. 22313001

[5] Xu KM, Smith GJ, Bahl J, Duan L, Tai H, Vijaykrishna D, Wang J, Zhang JX, Li KS, Fan XH, Webster RG, Chen H, Peiris JS, Guan Y 2007 The genesis and evolution of H9N2 influenza viruses in poultry from southern China, 2000 to 2005. J Virol 81:10389–10401. doi:10.1128/JVI.00979-07. 17652402 PMC2045440

[6] Xu KM, Li KS, Smith GJ, Li JW, Tai H, Zhang JX, Webster RG, Peiris JS, Chen H, Guan Y 2007 Evolution and molecular epidemiology of H9N2 influenza A viruses from quail in southern China, 2000 to 2005. J Virol 81:2635–2645. doi:10.1128/JVI.02316-06. 17192315 PMC1865985

[7] Bashashati M, Vasfi Marandi M, Sabouri F 2013 Genetic diversity of early (1998) and recent (2010) avian influenza H9N2 virus strains isolated from poultry in Iran. Arch Virol 158:2089–2100. doi:10.1007/s00705-013-1699-2. 23640582

